# Assessing the Burden of Mental Disorder Diagnosis and Racial Disparities in the Military Health System During the COVID-19 Pandemic

**DOI:** 10.1007/s40615-025-02494-7

**Published:** 2025-05-30

**Authors:** Christal Clements, Amanda Banaag, Miranda Lynn Janvrin, Raquel Martin, Jessica Jackson, Christian Coles, Tracey Pérez Koehlmoos

**Affiliations:** 1 Uniformed Services University of the Health Sciences, 4301 Jones Bridge Rd., Bethesda, MD 20814 USA; 2 The Henry M. Jackson Foundation for the Advancement of Military Medicine, Inc., 6720A Rockledge Dr., Bethesda, MD 20817 USA; 3 Tennessee State University, 3500 John A Merritt Blvd, Nashville, TN 37209 USA

**Keywords:** Healthcare disparities, Health inequities, Mental health, Mental health services, Military personnel

## Abstract

**Background:**

This study aims to assess whether there were changes in the burden of mental disorder diagnosis in active-duty service women (ADSW) during the COVID-19 pandemic and whether disparities in mental health services persist.

**Methods:**

We conducted a retrospective open cohort study on ADSW across the Military Health System (MHS) using data from the Military Health System Data Repository to analyze the changes in burden of mental disorder diagnosis in ADSW during the COVID-19 pandemic to provide insight into the need for behavioral health providers.

**Results:**

We identified a total of 325,147 ADSW from October 2016 to February 2020 and a total of 305,626 ADSW from March 2020 to September 2022. During the pre-COVID-19 period, 19.1% of ADSW had at least one mental disorder diagnosis compared to only 16.3% of ADSW during the COVID-19 period. Adjusted regression analyses revealed that non-Hispanic Black, Hispanic, and Asian/Pacific Islander ADSW are less likely to have a mental disorder diagnosis compared to non-Hispanic White ADSW; however, interaction testing revealed a significant interaction between race/ethnicity and rank, resulting in higher odds of a mental disorder diagnosis across all ranks, excluding “other” rank, among non-Hispanic Black and Hispanic ADSW, and higher odds in Asian/Pacific Islander and American Indian/Alaska Native ADSW in enlisted ranks.

**Conclusions:**

Our analysis indicates an increased burden of mental disorder diagnoses during the COVID-19 period; however, there was no disparity between mental health services received by Black ADSW and White ADSW overall during either the COVID-19 period or the pre-COVID-19 period, despite some variation by rank.

**Supplementary Information:**

The online version contains supplementary material available at 10.1007/s40615-025-02494-7.

## Introduction

Black women seeking mental health care in the United States experience significant disparities in both mental health outcomes and access to mental healthcare services due to their race. These conditions were further exacerbated by the COVID-19 pandemic [1, 2]. Black active-duty service women (ADSW) experience additional stressors related to their service and the requirements of their position that can have detrimental effects on their mental and emotional health, including combat stress, the stress of a minority status, military sexual violence, divorce, and parenting [3]. Research on mental health outcomes for Black ADSW indicate that they, like their civilian counterparts, seek mental health care at rates much lower than their White peers, despite having universal access to health care services through the Military Health System (MHS) [4]. The MHS guarantees universal healthcare coverage for approximately 9.6 million beneficiaries, of whom approximately 14% are active-duty service members from each of the Services, with the remainder being non-active-duty dependents and retirees. Approximately 17.5% of ADSW are women, and among those ADSW, 25.6% are Black [5, 6]. Yet research on racial disparities in mental health care among active-duty service members is limited and results have been inconsistent [7, 8].

The Armed Forces Health Surveillance Division (AFHSD) reported that from 2016 to 2020, 456,293 active component service members were diagnosed with at least one mental disorder [9]. Of those with a disorder, 43.8% met the diagnostic criteria for more than one illness, with many diagnoses attributable to adjustment disorder, depressive disorders, alcohol-related disorders, and post-traumatic stress disorder (PTSD) [9]. By gender, AFHSD reported that overall incidence rates of mental disorder diagnoses were higher in females than male service members.

Research prior to the COVID-19 pandemic has indicated that Black active-duty service members are less likely than White active-duty service members to use behavioral health services, but the pandemic has been reported to have disproportionately impacted the mental and physical health of Black Americans [2, 10]. This study aims to assess whether there were changes in the burden of mental disorder diagnosis in ADSW during the COVID-19 pandemic and whether disparities in mental health services persist.

## Methods

### Data Source and Study Population

Utilizing data obtained from the Military Health System Data Repository (MDR), we performed a retrospective open cohort study on female U.S. Army, Air Force, Navy, and Marine Corps active-duty service members over two study periods, October 1, 2016–February 28, 2020 (pre-COVID-19 period), and March 1, 2020–September 30, 2022 (COVID-19 period). The MDR contains administrative and healthcare claims for Military Health System beneficiaries, which include active duty service members, retirees, and their dependents [11]. The MHS operates as a bifurcated system, offering care through military treatment facilities (direct care) and civilian fee-for-service facilities via the Department of Defense TRICARE benefit (private sector care) [12]. TRICARE provides coverage for 9.6 million beneficiaries and does not include care delivered in theater (during deployment or war time operations) or through the Veteran’s Health Administration [13]. To ensure consistent access to care in the Military Health System (MHS), service women in the National Guard and Reserves were excluded from the study.

The primary outcome variable for this study was the prevalence of mental disorder diagnosis, which was identified using International Classification of Diseases, Tenth Revision (ICD-10), diagnostic codes. These codes were used to identify all women in our cohort with a diagnosis during the two study periods. Patient demographics, such as age, race and ethnicity, marital status, branch of service, and military rank as a proxy for socioeconomic status, were examined as predictive factors of a mental disorder diagnosis. The full list of ICD-10 mental disorder diagnostic codes used can be found in Online Resource 1.

### Statistical Analyses

Descriptive statistics were performed on study population demographics (age, marital status, branch of service, and rank), prevalence of mental disorder diagnosis expressed as per 100 ADSW, and chi-square tests to assess for significant differences in mental disorder diagnosis by all categorical variables. Unadjusted and adjusted logistic regressions with 95% confidence intervals were used to assess for the likelihood of mental disorder diagnosis among ADSW before and during the COVID-19 pandemic. Lastly, subsequent adjusted logistic regression analysis was performed to assess for the interaction between race and rank. Statistical significance was set at *p* < 0.05, and all analyses were performed using SAS 9.4. This study was determined to be exempt by the Institutional Review Board of the Uniformed Services University of the Health Sciences.

## Results

We identified a total of 326,147 ADSW from October 2016 to February 2020 and a total of 305,626 ADSW from March 2020 to September 2022. The pre-COVID-19 and COVID-19 populations were demographically similar, with both populations being predominantly non-Hispanic White, unmarried, and of Junior Enlisted rank (Table 1).

**Table 1:** Demographics of ADSW by mental disorder diagnosis status

	Pre-COVID-19 period		COVID-19 period	
	Total cohort	MH diagnosis	Total cohort	MH diagnosis
	*N* (col %)	*N* (col %)	*N* (col %)	*N* (col %)
Total	326,147 (100)	62,249 (100)	305,626 (100)	49,723 (100)
Age group				
18 to 24	185,738 (56.9)	28,817 (46.3)	160,405 (52.5)	21,557 (43.3)
25 to 34	99,447 (30.5)	21,165 (21.3)	101,381 (33.2)	17,532 (35.3)
35 to 44	33,542 (10.3)	9988 (29.8)	36,673 (12.0)	8891 (17.9)
45 to 54	6803 (2.1)	2140 (31.5)	6478 (2.1)	1601 (3.2)
55 +	617 (0.2)	139 (0.2)	689 (0.2)	142 (0.3)
Race				
Non-Hispanic White	144,648 (44.3)	28,360 (45.6)	129,442 (42.3)	21,345 (42.9)
Non-Hispanic Black	8257 (25.3)	17,446 (28.0)	73,652 (24.1)	13,595 (27.3)
Hispanic	58,871 (18.0)	9939 (16.0)	61,378 (20.1)	9035 (18.2)
Asian/Pacific Islander	22,935 (7.0)	3303 (5.3)	22,694 (7.4)	2729 (5.5)
American Indian/Alaskan Native	4925 (1.5)	1000 (1.6)	4254 (1.4)	716 (1.4)
Other	10,589 (3.2)	1934 (3.1)	13,329 (4.4)	2235 (4.5)
Missing	1632 (0.5)	267 (0.4)	877 (0.3)	68 (0.1)
Marital status				
Married	119,119 (36.5)	27,793 (44.6)	129,466 (42.4)	24,854 (50.0)
Unmarried	207,028 (63.5)	34,456 (55.3)	176,160 (57.6)	24,869 (50.0)
Rank				
Junior Enlisted	198,172 (60.8)	33,854 (54.4)	167,328 (54.7)	24,436 (49.1)
Senior Enlisted	74,022 (22.7)	20,698 (33.2)	82,013 (26.8)	18,427 (37.1)
Junior Officer	41,617 (13.8)	5948 (9.6)	42,905 (14.0)	5169 (10.4)
Senior Officer	4996 (1.5)	941 (1.5)	5656 (1.8)	975 (2.0)
Warrant Officer	1689 (0.5)	541 (0.9)	1769 (0.6)	448 (0.9)
Other	5650 (1.7)	267 (0.4)	5955 (1.9)	268 (0.5)
Branch of service				
Army	112,993 (34.6)	26,240 (42.1)	100,314 (32.8)	18,250 (36.7)
Air Force	91,342 (28.0)	16,195 (26.0)	88,927 (29.1)	13,486 (27.1)
Navy	95,666 (29.3)	15,938 (25.6)	92,504 (30.3)	14,611 (29.4)
Marine Corps	26,146 (8.0)	3876 (6.2)	23,881 (7.8)	3376 (6.8)

During the pre-COVID-19 period, 19.1% of ADSW had at least one mental disorder diagnosis compared to 16.3% during the COVID-19 period (Table 1). Similar overall decreases in the prevalence of a mental disorder diagnosis were observed across all racial-ethnic groups, by marital status, across all service branches, and across all military ranks (Table 1).

We calculated adjusted odds ratios (aOR) to determine which subgroups were at higher odds of receiving a mental disorder diagnosis. Compared to ADSW ages 18 to 24, all other age groups were at odds of receiving a mental disorder diagnosis during both the pre-COVID-19 and COVID-19 periods, with the likelihood increasing with age and decreasing from the pre-COVID-19 to the COVID-19 period within each age group (Table 2). When analyzed by race, non-Hispanic Black ADSW had a lower odds of a mental disorder diagnosis during the pre-COVID-19 period (aOR = 0.89, 95% CI = 0.87–0.91) compared to non-Hispanic White ADSW, but no significant difference in odds during the COVID-19 period (aOR = 0.98, 95% CI = 0.96–1.01) compared to White ADSW. All ranks, excluding “other” rank, had significantly higher odds of mental disorder diagnosis compared to Senior Officers, with the highest odds observed among Senior Enlisted ADSW during both periods and a decrease in odds across all rank groups from the pre-COVID-19 to the COVID-19 period (Table 2).

**Table 2:** Unadjusted and adjusted log-binomial regression analysis for risk of mental disorder diagnosis among the ADSW cohort

	Pre-COVID-19	COVID-19
	aOR (95% CI)	aOR (95% CI)
Age group (years)		
18 to 24 (ref)	1	1
25 to 34	1.32 (1.29–1.35)*	1.23 (1.19–1.26)*
35 to 44	2.11 (2.04–2.19)*	1.90 (1.83–1.97)*
45 to 54	2.82 (2.65–3.01)*	2.37 (2.21–2.54)*
55 +	2.46 (2.01–3.00)*	2.40 (2.21–2.54)*
Race code		
Non-Hispanic White (ref)	1	1
Non-Hispanic Black	0.89 (0.87–0.91)*	0.98 (0.96–1.01)
Hispanic	0.78 (0.76–0.80)*	0.82 (0.80–0.84)*
Asian/Pacific Islander	0.61 (0.58–0.63)*	0.62 (0.60–0.65)*
American Indian/Alaskan Native	0.98 (0.91–1.06)	0.93 (0.86–1.01)
Other	0.99 (0.94–1.04)	1.02 (0.97–1.07)
Marital status		
Married (ref)	1	1
Unmarried	0.80 (0.79–0.82)*	0.81 (0.79–0.83)*
Rank		
Junior Enlisted	2.35 (2.16–2.56)*	1.85 (1.70–2.01)*
Senior Enlisted	3.03 (2.80–3.29)*	2.29 (2.11–2.48)*
Junior Officer	1.26 (1.16–1.37)*	1.09 (1.01–1.18)*
Senior Officer (ref)	1	1
Warrant Officer	2.36 (20.7–2.68)*	1.91 (1.67–2.18)*
Other	0.57 (0.49–0.67)*	0.54 (0.47–0.64)*
Service		
Army (ref)	1	1
Air Force	0.65 (0.64–0.67)*	0.77 (0.75–0.79)*
Navy	0.65 (0.63–0.66)*	0.81 (0.80–0.84)*
Marine Corps	0.63 (0.60–0.65)*	0.81 (0.78–0.85)*

Adjusted by all variables included in the models: age group, race/ethnicity, marital status, and service

*aOR* adjusted odds ratio, *CI* confidence interval

*Statistically significant, *p* < 0.05

Subsequent regression analysis with interaction tests between race/ethnicity and rank revealed that non-Hispanic Black and Hispanic women of all ranks, excluding “other” rank and Hispanic women in a Senior Officer rank, were at a higher risk for mental disorder diagnosis during both periods compared to White ADSW in a Senior Officer rank (Table 3), and American Indian/Alaska Native and Asian/Pacific Islander ADSW in enlisted ranks had higher odds of a mental disorder diagnosis during both COVID-19 periods (Table 3).

**Table 3:** Adjusted log-binomial regression analysis with race and rank interaction

	Pre-COVID-19 period	COVID-19 period
	aOR (95% CI)	aOR (95% CI)
Race*rank interaction		
Non-Hispanic White, Senior Officer (ref)		
Non-Hispanic White, Junior Enlisted	2.81 (2.54–3.10)*	2.31 (2.09–2.56)*
Non-Hispanic White, Senior Enlisted	1.39 (1.35–1.43)*	2.07 (1.90–2.26)*
Non-Hispanic White, Junior Officer	1.25 (1.13–1.38)*	1.12 (1.01–1.24)*
Non-Hispanic White, Warrant Officer	1.95 (1.60–2.39)*	1.61 (1.30–2.00)*
Non-Hispanic White, Other	0.50 (0.41–0.62)*	0.56 (0.46–0.68)*
Non-Hispanic Black, Junior Enlisted	2.22 (2.01–2.46)*	1.73 (1.58–1.89)*
Non-Hispanic Black, Senior Enlisted	3.13 (2.84–3.46)*	2.63 (2.38–2.91)*
Non-Hispanic Black, Junior Officer	1.83 (1.64–2.06)*	1.73 (1.54–1.95)*
Non-Hispanic Black, Senior Officer	1.41 (1.17–1.70)*	1.82 (1.52–2.17)*
Non-Hispanic Black, Warrant Officer	2.11 (2.58–3.74)*	2.83 (2.34–3.43)*
Non-Hispanic Black, Other	0.81 (0.54–1.21)	0.52 (0.32–0.85)*
Hispanic, Junior Enlisted	1.95 (1.76–2.16)*	1.68 (1.51–1.86)*
Hispanic, Senior Enlisted	2.89 (2.61–3.21)*	2.32 (2.09–2.58)*
Hispanic, Junior Officer	1.35 (1.18–1.54)*	1.24 (1.09–1.42)*
Hispanic, Senior Officer	1.62 (1.21–2.16)*	1.12 (0.84–1.50)
Hispanic, Warrant Officer	2.00 (1.48–2.70)*	2.12 (1.61–2.80)*
Hispanic, Other	0.86 (0.58–1.28)	0.83 (0.53–1.30)
Asian/Pacific Islander Junior Enlisted	1.55 (1.38–1.73)*	1.31 (1.16–1.47)*
Asian/Pacific Islander, Senior Enlisted	2.00 (1.78–2.24)*	1.58 (1.41–1.78)*
Asian/Pacific Islander, Junior Officer	1.03 (0.90–1.18)	0.96 (0.83–1.11)
Asian/Pacific Islander, Senior Officer	0.82 (0.61–1.10)	1.02 (0.78–1.33)*
Asian/Pacific Islander, Warrant Officer	1.92 (1.26–2.94)*	0.98 (0.61–1.57)
Asian/Pacific Islander, Other	0.8 (0.63–1.24)	0.62 (0.43–0.90)*
American Indian/Alaskan Native, Junior Enlisted	2.81 (2.45–3.23)*	2.01 (1.71–2.37)*
American Indian/Alaskan Native, Senior Enlisted	3.06 (2.63–3.56)*	2.46 (2.10–2.87)*
American Indian/Alaskan Native, Junior Officer	1.31 (1.02–1.68)*	1.24 (0.97–1.59)
American Indian/Alaskan Native, Senior Officer	1.77 (0.85–3.69)	1.41 (0.67–2.97)
American Indian/Alaskan Native, Warrant Officer	3.66 (1.56–8.59)*	1.75 (0.57–5.42)
American Indian/Alaskan Native, Other rank	0.46 (0.11–1.89)	0.47 (0.11–1.94)
Other, Junior Enlisted	2.82 (2.50–3.18)*	2.33 (2.06–2.63)*
Other, Senior Enlisted	3.14 (2.76–3.57)*	2.61 (2.32–2.95)*
Other, Junior Officer	1.32 (1.09–1.58)*	1.24 (1.05–1.45)*
Other, Senior Officer	0.87 (0.50–1.51)	1.04 (0.72–1.49)
Other, Warrant Officer	3.07 (1.50–6.30)*	4.53 (2.10–9.80)*
Other race, other rank	0.35 (0.15–0.79)*	0.95 (0.62–1.47)

Adjusted by main effects: age group, marital status, and service

*aOR* adjusted odds ratio, *CI* confidence interval

*Statistically significant, *p* < 0.05

**Fig. 1:**
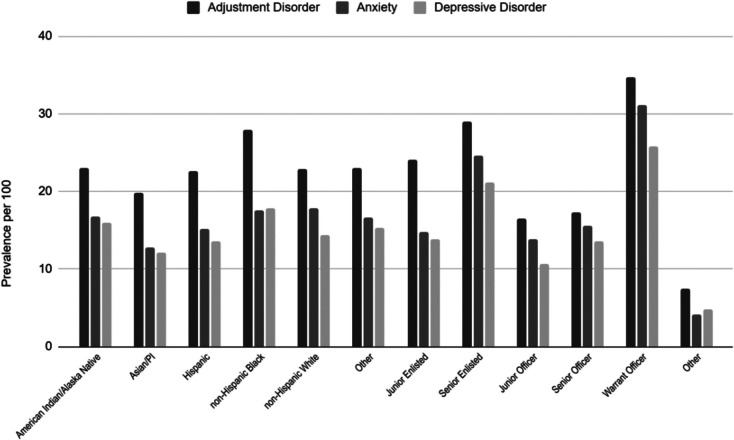
Top three mental disorder diagnoses for ADSW across all service branches by race and rank during the pre-COVID-19 period

**Fig. 2:**
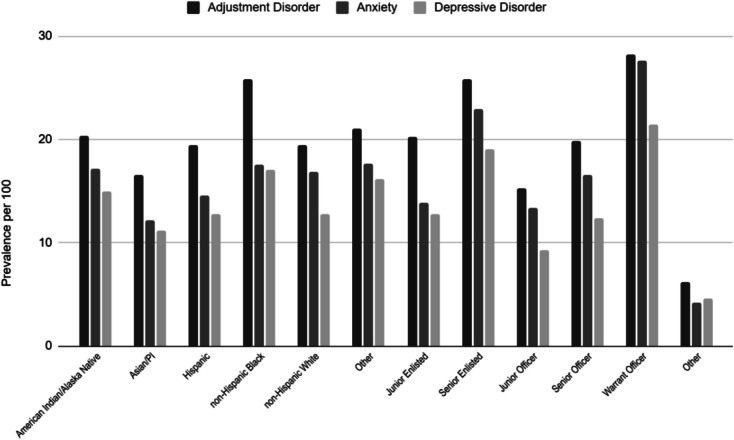
Top three mental disorder diagnoses for ADSW across all service branches by race and rank during the COVID-19 period

The three most frequent mental disorder diagnoses during both the pre-COVID-19 and COVID-19 periods were adjustment disorder, anxiety, and depressive disorders (Table 4). During both periods, adjustment disorder comprised the majority of diagnoses among all ADSW, regardless of both race and rank (Figs. 1 and 2).

**Table 4:** Mental disorder diagnoses by frequency

	Pre-COVID-19 period		COVID-19 period	
Mental disorder diagnosis category	*N*	% of cohort	*N*	% of cohort
Adjustment disorder	77,766	23.8	63,665	20.8
Anxiety	54,862	16.8	49,507	16.2
Depressive disorders	48,725	14.9	42,275	13.8
PTSD	18,254	5.6	18,420	6.0
Alcohol use disorder	9937	3.0	7222	2.4
Suicidal ideations	8415	2.6	7275	2.4
Personality	5290	1.6	4089	1.3
Bipolar	2076	0.6	2235	0.7
Substance use	1603	0.5	990	0.3
Psychotic	1135	0.3	926	0.3
Schizophrenia	281	0.1	232	0.1

## Discussion

Our analysis of changes in the burden of mental disorder diagnosis in ADSW before and during the COVID-19 pandemic provided insight into the need for behavioral health providers within the MHS. In our analysis of changes in the burden of mental disorder diagnosis in ADSW during the COVID-19 pandemic, we identified a total of 326,147 ADSW from October 2016 to February 2020 and a total of 305,626 ADSW from March 2020 to September 2022, of whom 19.1% had at least one mental disorder diagnosis in the pre-COVID-19 period and 16.3% had at least one mental disorder diagnosis during the COVID-19 period. The decrease in ADSW with a diagnosed mental disorder condition may be due to ADSW experiencing reduced access to mental health services during the COVID-19 period as a result of the pandemic. This finding aligns with a report from the American Hospital Association indicating a substantial decrease in behavioral health disorder services in the U.S. for all ages during this same period [14]. It is unlikely that this finding indicates a reduction in mental health conditions during the COVID-19 period, as research within the general population indicates increases in symptoms of stress, depressive disorders, and generalized anxiety disorder alongside increases in reports of experiencing symptoms of these disorders, but not receiving counseling or therapy [15, 16].

Black Americans are less likely to seek treatment for mental health conditions, due in part to a broad sense of cultural mistrust in the medical system and compounded by historical mistreatment of Black patients in healthcare settings, which has led to the stigmatization of mental health treatment within the Black community [17, 18]. A qualitative study of Black mental health consumers on exposure to stigmatizing views regarding mental health indicated that respondents avoided mental health treatment for fear of being seen as weak, perceived damage to family reputation, and concerns about social judgement and rejection if they pursued treatment [19]. Black women face other unique challenges in seeking mental health care. A qualitative study of Black women looking at barriers when seeking mental health services indicated knowledge of services, cultural perception, discrimination, and lack of awareness as being individual barriers to seeking treatment [20]. This can lead to delayed care or failure to seek care entirely and increase Black women’s vulnerability to depressive symptoms associated with stress [21]. Despite this, our findings indicate that there was essentially no difference between Black and White ADSW in terms of receiving a mental disorder diagnosis during the COVID-19 pandemic. This is in contrast to findings from both a 2023 survey that indicated that Black adults are less likely than White adults to have received mental health services in the preceding three years as well as a narrative review that indicated that Black youth were overall less likely to utilize mental health services during the pandemic [22, 23]. The universal healthcare coverage received by ADSW may be mitigating this disparity [8].

In our analysis, we used military rank as a proxy for socioeconomic status. Rank is often used as a proxy for socioeconomic status, in studies of the military population, as increased rank is associated with increased education and income [24]. Our findings suggest that Junior Enlisted, Senior Enlisted, and Junior Officer ADSW were at increased risk of receiving a mental disorder diagnosis compared to Senior Officer ADSW during both the pre-COVID-19 and COVID-19 periods, with the highest risk being among both Junior and Senior Enlisted ADSW. This indicates that Service women of a lower socioeconomic status were more likely to receive a mental disorder diagnosis during both time periods. This finding is in line with findings from McKibben et al. that indicates that the rates of women and enlisted soldiers to use mental health services were 39 and 93% higher than those of other members in the active-duty Army population [25] as well as findings from Kivimäki et al. that indicate that individuals with a lower socioeconomic status were at increased risk of experiencing mood and psychotic disorders, among other conditions [26].

This study had several strengths. The use of the electronic medical record for our assessment of mental disorder diagnoses among ADSW allowed for all formal diagnoses within the MHS during both time periods to be included in this study. The individual-level data and large sample size of this assessment allowed for racial subgroup analysis with sufficient power to make statistically significant comparisons.

This study also had some limitations. We were unable to include data on ADSW who sought mental health services outside of MHS through sources like Military One Source or self-paying. This study utilized secondary healthcare claims data which carries the potential for inaccurate coding and underestimation of diseases and disorders. Additionally, as our dataset did not have an education level, variable rank was used as a close, but imperfect proxy.

Future studies should explore the long-term impacts of the COVID-19 pandemic on mental disorder diagnoses among active-duty service women, identify barriers to mental health services both during and after the COVID-19 pandemic, and examine the role of race and rank in mental health risk and diagnoses. Additional research is also needed investigating provider diversity across the military branches and the potential impact on service members. While investigating provider diversity, it would also be beneficial to address the types of education that are provided to mental health providers as well as the therapeutic orientations that are utilized with patients.

## Conclusion

The purpose of this study was to analyze the changes in the burden of mental disorder diagnosis in ADSW during the COVID-19 pandemic to provide insight into the need for behavioral health providers within the MHS and to determine if disparities in mental health services persist. Our findings indicate an increase in mental disorder burden during the pandemic, with 19.1% of ADSW having had at least one mental disorder diagnosis compared to only 16.3% of ADSW during the COVID-19 period. This increase in mental disorder burden, if it persists past the COVID-19 pandemic period, indicates that the MHS may need additional behavioral and mental health providers. Additionally, we found no differences in mental disorder diagnosis between Black and White ADSW either prior to or during the COVID-19 period. It is possible that the universal healthcare coverage received by ADSW may be mitigating this disparity. Taken together, these results indicate that although the burden of mental disorder diagnosis increased during the COVID-19 pandemic period, racial disparities in overall mental health care between Black and White ADSW were mitigated.

## Supplementary Information

Below is the link to the electronic supplementary material.

ESM 1 (DOCX 13.8 KB)

## Data Availability

The data that support the findings of this study are available from the United States Defense Health Agency. Restrictions apply to the availability of these data, which were used under Federal Data User Agreements for the current study, and so are not publicly available.

## Declarations

### Disclaimer

The contents of this publication are the sole responsibility of the authors and do not necessarily reflect the views, assertions, opinions, or policies of the Uniformed Services University of the Health Sciences (USUHS), the Henry M. Jackson Foundation for the Advancement of Military Medicine, Inc. (HJF), the Department of Defense (DoD), or the Departments of the Army, Navy, or Air Force. Mention of trade names, commercial products, or organizations does not imply endorsement by the U.S. Government.
