# Supplementary material for: Assessing the Burden of Mental Disorder Diagnosis and Racial Disparities in the Military Health System During the COVID-19 Pandemic
Source: J Racial Ethn Health Disparities. 2025 May 30;13(4):3235–42. doi: 10.1007/s40615-025-02494-7 (PMC13346269; doi:10.1007/s40615-025-02494-7)
Supplement: Supplementary file 1 — (DOCX 13.8 KB) [file 40615_2025_2494_MOESM1_ESM.docx]

**Supplemental Table 1. List of ICD-10 Codes**

**ICD-10 Code Long description**

Adjustment disorders (F43.2*, F43.8, F43.9, F93.0, F94.8, F94.9)

Alcohol-related disorders (F10.1*, F10.2*)

Substance-related disorders (F11.2*, F12.2*, F13.2*, F14.2*, F15.2*, F16.2*, F18.2*,

F19.2*,F11.1*, F12.1* F13.1*, F14.1*, F15.1*, F16.1*, F18.1*, F19.1*)

Anxiety disorders (F40.*, F41.*, F42.*)

Post-traumatic stress disorder (F43.1*)

Depressive disorders (F32.*, F33.*, F34, F34.1, F34.8, F34.9, F39, F348.1, F34.89)

Bipolar disorder (F30.*, F31.*, F34.0)

Personality disorders (F21, F60.*)

Schizophrenia (F20*, F25*)

Psychotic disorders (other psychoses) (F06.0, F06.2, F22–F24, F28, F29)

Ideation, suicidal (R45.851)

Other mental health disorder (Any other code between F01–F99 (excluding F07.81, F70–F79,

F17.*, F80.*–F82.*, F84.*, F88–F89))
